# Knowledge, Attitude, and Practice of Dutch Dentists on Oral Leukoplakia and Their Possible Role in Its Follow-Up

**DOI:** 10.1016/j.identj.2024.10.021

**Published:** 2025-01-03

**Authors:** Ahmad M. Najim, Josef J.M. Bruers, Jan GAM de Visscher

**Affiliations:** a Department of Oral and Maxillofacial Surgery/Oral Pathology, Amsterdam University Medical Centre, Location Vrije Universiteit and Academic Centre for Dentistry Amsterdam (ACTA), Amsterdam, The Netherlands; b Department of Oral Public Health, Academic Centre for Dentistry Amsterdam (ACTA), University of Amsterdam and Vrije Universiteit Amsterdam, Amsterdam, The Netherlands; c Royal Dutch Dental Association (KNMT), Utrecht, The Netherlands

**Keywords:** Oral leukoplakia, oral squamous cell carcinoma, Dutch dentists, knowledge, attitude, follow-up

## Abstract

**Objective:**

To assess the knowledge, attitude, and practice of Dutch dentists on oral leukoplakia (OL) and to what extent these aspects are related to whether or not dentists regularly monitor patients with OL.

**Material and methods:**

A self-developed questionnaire was distributed via a web survey among a sample of dentists participating in an intervision program. Of 1626 invited dentists, 437 (26.9%) answered the questionnaire; 52.6% were females and 47.4% males, 36.4% were 44 years or younger, 24.0% were 45 to 54 years old and 39.6% were 55 years or older, 94.1% were general dental practitioners, 60.2% were practice owners, and 49.9% work more than 31 hours per week.

**Results:**

In total, 57.0% performed regular follow-up of patients with OL. Compared to dentists who do not perform regular follow-up of patients with OL, those who do were more confident in performing control, were more likely to be practice owners, checked a greater number of oral subsites during dental check-ups, had encountered more cases of suspected oral cancer among their own patients, and were more likely to implement effective policies when faced with mucosal lesions without a clear diagnosis. The vast majority of all participants indicated that their knowledge about oral mucosal diseases, OL, and oral cancer is limited. Just over half reported receiving insufficient education on these topics during their dental study.

**Conclusions:**

It seems that interested dentists who have an affinity for oral mucosal diseases can properly fulfil the controlling and detecting role on selected patients with OL. Additional education on various aspects of OL is required.

**Clinical relevance:**

In the Netherlands, interested dentists can responsibly follow up on a large proportion of patients with OL.

## Introduction

Oral leukoplakia (OL) is defined as a predominantly white plaque of questionable risk, having excluded (other) known diseases or disorders that carry no increased risk for cancer.^1^^,^^2^ OL is the most common oral potentially malignant disorder (OPMD), with an estimated global prevalence of 1.39%, varying from 0.12% to 33.33%.^3^ The overall estimated prevalence rates of OL in Europe in population-based, clinic-based, and specific population studies are 1.82%, 0.38%, and 4,85%, respectively.^3^ In two systematic reviews, the worldwide pooled rate of malignant transformation (MT) of OL to an oral squamous cell carcinoma (OSCC) was between 7.2% and 9.8%, but there are wide regional differences of MT varying from 0.13% to 40.8%.^4^, ^5^, ^6^, ^7^ The reported annual MT rates of OL range from 1.36% to 2.6%.^8^^,^^9^ We have recently reported a constant annual MT rate of 4.9% in our cohort of OL patients with a long follow-up.^10^ The large global variations in the prevalence and MT rates of OL may be attributed to the variation in used criteria and definition for OL diagnosis, worldwide geographical location, differences in the exposure and extent of use of etiological factors, composition of study population (population or hospital based), study design (prospective or retrospective), whether the composition of the cohort of patients is constant, and length of follow-up. Various clinical features, histopathological diagnosis, molecular biomarkers, and genetic changes may be, independent or in combination, predictive for an increased risk of MT of OL.^5^^,^^7^^,^^11^^,^^12^ Until now, the presence and degree of epithelial dysplasia is the most significant independent risk factor for MT, although OL without dysplasia can also become malignant.^7^^,^^10^ However, these potential predictors of MT are only partly useful and may provide some information in a series of patients but are certainly not accurate enough for predicting MT of OL in the individual patient. Removal of the clinical visual OL lesion by either surgical excision, CO_2_-laser evaporation, or a combination of both modalities not only shows high recurrence rates but also does not prevent MT.^13^^,^^14^ MT of OL may occur many years after the initial clinical and histopathological diagnosis and therefore long-term or even lifelong follow-up of patients with treated and untreated OL is generally recommended whereby the regular intervals vary from 6 to 12 months. The main reason for periodic controls is the early detection of possible MT of OL or of OSCC elsewhere in the oral cavity. This is of importance since treatment of early OSCC is associated with high survival rates, limited treatment-related side effects, and better quality of life outcomes.^15^^,^^16^ Follow-up of OL is usually performed at specialised departments such as oral medicine, oral and maxillofacial surgery, and head and neck surgery. Although the percentage of MT of OLs without dysplasia is low, in most centres these patients will also remain in periodic follow-up. Long-term or even lifelong follow-up of OL results in an ever-increasing number of patients, is time-consuming, and poses an increasing burden on healthcare professionals and the healthcare system and an increase in healthcare costs. The WHO also indicates that the healthcare workforce is under pressure due to a high percentage of staff nearing retirement age in the next decade, with inadequate recruitment and retention exacerbating the issue.^17^ Furthermore, healthcare costs in Europe are rising and are expected to increase by 0.8% annually under the current circumstances.^18^ To reduce the burden, after the definitive clinical and histopathological diagnosis of OL at a specialised centre, follow-up of OL patients could possibly be performed by their dentist during regular dental check-ups using the same follow-up protocol as at the hospital. However, a recent study reported that regular follow-up at a specialised centre for patients with OPMDs, consisting mainly of patients with OL, led to earlier detection of OSCC compared to a group of patients that were referred back to their general dentists. Although dentists were instructed on the follow-up strategy, their patients presented with notably larger tumour sizes.^15^ This is in contrast with our finding in a cohort of patients with OL, where there was no statistically significant difference in clinical tumour size between patients that had their regular follow-up at the outpatient clinic and those who had follow-up conducted by their dentist.^19^ The aim of the current study is to assess the knowledge, attitude, and practice of Dutch dentists on OL and to what extent these aspects are related to whether or not dentists regularly monitor patients with OL.

## Materials and methods

This study was conducted as a survey among a sample of dentists in the Netherlands. A self-constructed survey was conducted and distributed digitally by the Royal Dutch Dental Association (KNMT) to a sample of dentists who take part in an intervision program set up by the KNMT. The design of this study was submitted to and approved by the Ethical Review Committee (ETC) of the Academic Centre for Dentistry Amsterdam (ACTA), under application number 2024-56118.

### Research instrument

For the purposes of this study, a questionnaire was constructed, which consisted of 35 items divided into 4 sections. In the first section, 4 questions were asked about the work and practice situation of dentists. The second section consisted of 8 questions regarding their previous clinical experiences with patients with OL and OSCC and the third section consisted of 3 questions regarding views on the treatment of OL and OSCC. One of those questions involved 13 Likert-type items. In the fourth section, 5 clinical cases with oral mucosal lesions were presented and illustrated with light photos, prompting dentists in 4 questions for each case to discern the most probable diagnosis, recommend the most appropriate treatment strategy, and express their confidence levels regarding their chosen course of action. The questionnaire has been tested for content validity and face validity by multiple experts on the topic.

### Data collection

A convenience sample of 1626 dentists, all of whom participated in intervision program ‘IQual’, were invited in October 2023 to fill in the questionnaire by an independent research agency (KBA Nijmegen) on behalf of the KNMT through email. The email contained a personalised link to the online questionnaire. Reminders were sent after 3 and 6 weeks.

### Data processing

The independent research agency processed the raw data from the questionnaires and compiled an encrypted database. Various known background characteristics of the respondents, such as years of experience, alma mater, and workplace region, were added anonymously to this database. Due to the encrypted database, responses were not able to be linked back to individual dentists. Only the encrypted database, in which individual dentists and practices were completely unrecognisable, was handed over to the researchers.

### Statistical analysis

For the 13 Likert-type items regarding views on the treatment of OL and OSCC, it was explored whether there were any related aspects. This was done using principal component analysis with Varimax rotation, where an eigenvalue of 1 was used to determine the number of factors. This led to 2 components, which together explained 48.2% of the variance. In the first component, 6 items were combined and expressed *‘acquired knowledge about leukoplakia through good education’* (Appendix Table J, items a, b, c, d, l, and m from Appendix Table I). The second component consisted of 3 items, expressing *‘confidence to take over the follow-up of patients with OL from an oral and maxillofacial surgeon’* (Appendix Table K, items e, f, and g from Appendix Table I). A sum score was determined for both components and a reliability analysis was performed. The Cronbach's alpha for the component ‘*confidence to check for leukoplakia’* was 0.889, and for the component ‘*knowledge about leukoplakia through good education’* was 0.798, which in both cases indicated good internal consistency. The sum score for the first component ranged from 3 (very little) to 15 (very much) and that of the second component from 6 (very little) to 30 (very much).

The group of respondents was described using descriptive statistical measures. For the univariate comparison with regard to personal and professional characteristics between dentists who do and dentists who do not monitor leukoplakia patients at the request of an oral and maxillofacial surgeon (OMFS), the Chi-square test or the independent *t* test was used. Subsequently, multiple logistic regression was used to determine which of these characteristics can explain whether or not respondents monitor leukoplakia patients at the request of an OMFS. For this purpose, only those characteristics that showed a *P* value of .10 or lower in the univariate analysis were used. The final model was determined after stepwise elimination of nonsignificant characteristics, which provided a significantly better estimate than the baseline intercept (*P* < .001). The ‘goodness of fit test’ for the model showed no significant deviation (*P* = .543) from the null hypothesis that the model is a ‘good enough’ fit to the data. All analyses were performed using Statistical Package for Social Sciences (SPSS) for Windows, version 28.

## Results

### Research group

Ultimately, 437 (26.9%) of 1626 invited dentists answered the questionnaire. Of these dentists, 52.6% were female and 47.4% male, while 36.4% were 44 years or younger, 24.0% were 45 to 54 years old, and 39.6% were 55 years or older. In addition, most of the respondents (94.1%) were general dental practitioners and 5.9% only worked as dental specialists (such as implantology, endodontics, periodontics), while 60.2% of the respondents were practice owners. Furthermore, 11.9% were active up to 20 hours per week treating patients, 38.2% were active up to 21 to 30 hours, and 49.9% were active up to 31 or more hours per week (Table 1).

**Table 1:** General and occupation-specific characteristics of dentists in relation to whether or not performing follow-up of patients with OL at request of OMFS.

	Yes	No	Total	*P*
*Gender*				**.012**
Male	52.6%	40.4%	47.4%	
Female	47.4%	59.6%	52.6%	

*Age (y)*				**<.001**
34 or younger	6.8%	17.0%	11.2%	
35–44	24.1%	26.6%	25.2%	
45–54	24.5%	23.4%	24.0%	
55–64	34.2%	26.1%	30.7%	
65 or older	10.4%	6.9%	8.9%	

*Mean age (SD)*	*51.4 (11.0)*	*47.3 (11.9)*	*49.6 (11.6)*	***<.001***

*Graduation city*				.807
Amsterdam	29.7%	30.8%	30.2%	
Groningen	18.1%	16.0%	17.2%	
Nijmegen	36.2%	39.4%	37.5%	
Utrecht	10.8%	8.0%	9.6%	
Abroad	5.2%	5.8%	5.5%	

*Region practice location*				.521
South	33.7%	33.5%	33.6%	
West	28.9%	28.2%	28.6%	
East	26.9%	31.4%	28.9%	
North	10.5%	6.9%	8.9%	

*Active patient treatment*				**<.001**
Yes, as a practice owner	67.5%	50.5%	60.2%	
Yes, not as a practice owner	32.5%	49.5%	39.8%	

*Practice activities*				**.006**
General practitioner	85.5%	78.2%	82.4%	
General practitioner and dental specialist*	11.7%	11.7%	11.7%	
Dental specialist†	2.8%	10.1%	5.9%	

*Hours per week active patient treatment*				.073
20 or less	12.1%	11.7%	11.9%	
21–30	33.7%	44.1%	38.2%	
31 or more	54.2%	44.2%	49.9%	

*n*	249	188	437	

⁎ Dentist-implantologist (22×), restorative dentist (16×), dentist for orthodontics (7×), dentist sleep medicine (5×), dentist-pedodontologist (3×), anxiety dentist (3×), dentist-geriatrics (3×), dentist disability care (2×), dentist-gnathologist (2×), dentist maxillofacial prosthetics (1×), unknown (1×).

† Dentist-periodontist (8×), dentist-implantologist (5×), dentist disability care (4×), dentist-endodontist (3×), dentist-pedodontologist (3×), anxiety dentist (3×), dentist-geriatrics (2×), dentist-gnathologist (1×), restorative dentist (1×), dentist for orthodontics (1×), dentist sleep medicine (1×), dentist maxillofacial prosthetics (1×).

### Clinical practice

At least 9 out of 10 (89.5%) dentists indicated that they usually or always checked gingiva, cheek, vestibule, palate, tongue, and lip during regular dental check-up. For floor of mouth (79.4%) and especially tonsillar pillar (34.6%) these percentages were lower (Appendix Tables A and B). More than half (57.0%) of the dentists surveyed indicated to perform follow-up of patients with OL at the explicit request of an OMFS (Appendix Table C). When asked about their policy regarding a patient with an oral mucosal lesion without a clear clinical diagnosis 32.7% selected the right policy, ie, refer to or consult with an OMFS (Appendix Table D). The majority (86.7%) of the dentists in this study stated that they have had patients with suspected oral cancer. Almost all of these dentists referred the most recent of these patients to an OMFS, and in more than three-quarters of cases (62.7% vs 21.3%) it appeared to actually be oral cancer, whereby the diagnosis was confirmed by histopathological examination of a biopsy performed by the OMFS (Appendix Table E). In the cases in which it turned out to be oral cancer, the referring dentists were certain that it was oral cancer in many more cases than in the cases in which it turned out not to be oral cancer (80.3% vs 16.1%) (Appendix Table F). Furthermore, most dentists surveyed were able to correctly define the term oral epithelial dysplasia as the presence of abnormal cells (84.3%) and were able to correctly ascertain that OL of the floor of mouth and tongue shows a higher risk of MT than OL of the palate and gingiva (73.9%) (Appendix Tables G and H).

### Confidence

Most dentists (79.9%) indicated that they would need further education on oral mucosal diseases if they were to take over the follow-up of patients with leukoplakia (Appendix Table I). At the same time almost half (42.8%) of the dentists stated to be confident about knowledge and received education about oral mucosal diseases, leukoplakia, and oral cancer, while in this regard, 11.8% admitted to lack confidence (Appendix Table J). Furthermore, 20.1% of the dentists surveyed were confident and 41.2% were not confident in taking over the follow-up of patients with OL at different oral subsites and different histopathological diagnoses from an OMFS (Appendix Table K).

### Closer look at performing follow-up of patients with OL

Table 1 shows that various general and professional-specific characteristics are univariately associated with whether or not dentists perform follow-up patients with OL at the request of an OMFS. From Table 2 it becomes clear that these two groups of dentists also differ univariately with regard to various aspects of clinical practice and confidence of dentists.

**Table 2:** Occupation-specific characteristics of dentists in relation to whether or not performing follow-up of patients with OL at request of OMFS.

	Yes	No	Total	*P*
Number of oral subsites that are regularly checked (max. 8)*^,^†				**<.001**
6 or less	18.9%	36.2%	26.3%	
7–8	81.1%	63.8%	73.7%	

*Mean number (SD)*	*6.9 (1.4)*	*6.5 (1.6)*	*6.7 (1.5)*	***.001***

*Policy in case of mucosal lesion without a clear clinical diagnosis*				**.050**
Refer to/consult specialist	36.5%	27.7%	32.7%	
Wait and see	63.5%	72.3%	67.3%	

*Previously seen own patient with suspicion of oral cancer*†^,^‡				**.003**
No	8.0%	20.2%	13.3%	
Yes, referred, turned out to be oral cancer	66.3%	58.0%	62.7%	
Yes, referred, turned out not to be oral cancer	22.9%	19.1%	21.3%	
Yes, not referred, unknown if it was oral cancer	2.8%	2.7%	2.7%	

*Confident to perform regularly follow-up of patient with leukoplakia*§				**<.001**
No	70.3%	91.8%	79.9%	
Yes	29.7%	8.2%	20.1%	

*Confident because gathered knowledge and education about oral cancer*§				.196
No	54.2%	60.8%	57.2%	
Yes	45.8%	39.2%	42.8%	

*If I take over follow-up of patients with leukoplakia, I will also show patient to fellow dentist in practice*				.763
No	44.8%	43.3%	44.1%	
Yes	55.2%	56.7%	55.9%	

*If I take over follow-up of patients with leukoplakia, I do need further education about oral mucosal diseases*				**<.001**
No	27.4%	11.1%	20.1%	
Yes	72.6%	88.9%	79.9%	

*Leave follow-up of patients with leukoplakia to colleague who has experience and affinity with disease*				.701
No	90.1%	88.9%	89.6%	
Yes	9.9%	11.1%	10.4%	

*All patients with leukoplakia should be checked by an OMFS*				**.007**
No	68.9%	55.6%	62.9%	
Yes	31.1%	44.4%	37.1%	

*Defining the term oral epithelial dysplasia*¶				.232
Wrong	13.7%	18.2%	15.7%	
Right	86.3%	81.8%	84.3%	

*Estimate risk of malignant transformation of oral leukoplakia*║				.867
Wrong	25.7%	26.5%	26.1%	
Right	74.3%	73.5%	73.9%	

*n*	210–249	170–188	380–437	

⁎ Number of oral subsites (lip, cheek, vestibule, gingiva, tongue, floor of mouth, palate and tonsillar pillar; total = 8) that are always checked during periodic dental examination.

† *n =* 437.

‡ Combination of question ‘referred own patient with clinical suspicion of oral cancer’ and question ‘whether it turned out to be cancer or not’.

§ *n =* 383.

¶ *n =* 381.

║ *n =* 380.

Nevertheless, it is striking that a substantial proportion of those who say they monitor OL patients indicate that actually they are not confident to perform regular follow-up of these patients (70.3%), have insufficient knowledge about oral cancer (54.2%), need further education about oral mucosal diseases (72.6%), and believe that all these patients should be checked by an OMFS (31.1%). Moreover, 1 in 4 of them does not properly assess the risk of malignant transformation of OL (25.7%), and several do not provide a correct definition of the term oral epithelial dysplasia (13.7%).

Table 3, in which the results of the multivariate analysis are presented, shows that dentists who perform follow-up of patients with OL, compared to colleagues who do not, expressed confidence more often in following up with patients with leukoplakia (OR = 1.297, *P* < .001), indicated less often that they need further education on oral mucosal diseases if they should take over follow-up of patients with leukoplakia (0.433, *P* = .007), were more often practice owner (OR = 3.819, *P* = .014), have more often seen own patients with suspected oral cancer (OR = 2.371, *P* = .014), and implement good policies more often in case of a mucosal lesion without a clear diagnosis (OR = 1.773, *P* = .019), and they check more oral subsites during a dental check-up (OR = 1.174, *P* = .030).

**Table 3:** Multivariate relationship between whether or not to perform follow-up of patients with OL and general and occupational-specific characteristics of the dentists.

	Odds ratio	CI for odds ratio	*P*
Confident to follow-up patients with leukoplakia*	1.297	1.184–1.421	**<.001**
If I take over follow-up of patients with leukoplakia, I do need further education on oral mucosal diseases	0.433	0.235–0.797	**.007**
Practice owner†	3.819	1.390–11.144	**.014**
Seen own patient with suspicion of oral cancer	2.371	1.188–4.731	**.014**
Policy in case of mucosal lesion without a clear diagnosis§	1.773	1.098–2.865	**.019**
Number of oral subsides that are always checked during RDC	1.174	1.015–1.357	**.030**

*n* = 383.

Omnibus tests of model coefficients: Chi-square = 77.604, df = 6, *P* < .001.

Nagelkerke pseudo-R-Square = 0.245.

Hosmer & Lemeshow test: Chi-square = 3.204, df = 8, *P* = .921.

⁎ Scale score with minimum 3 and maximum 15.

† Dummy: practice owner(1) vs non-practice owner (0). ^#^ Dummy: seen before(1) vs never seen before (0).

§ Dummy: refer to specialist (1) vs wait and see (0). CI, confidence interval; RDC, regular dental check-up.

## Discussion

The aim of the present study was to assess the knowledge, attitude, and practice of Dutch dentists on OL and to what extent these aspects are related to whether or not dentists actually regularly follow up with patients with OL during routine dental check-up. It was striking that more than half (57%) of the respondents already monitored patients with OL, which was more than was expected in advance. These dentists demonstrated that they have knowledge of oral mucosal diseases in relatively more cases and are relatively more confident that they can actually monitor OL patients. At the same time, those who said they monitor OL patients indicated in quite many cases that they are actually not confident in performing regular follow-up of these patients. In addition, they demonstrated insufficient knowledge of oral mucosal diseases. The question arises to what extent these dentists feel that they are qualified to perform regular follow-up of OL patients and if they may overestimate themselves. However, it is also debatable whether extensive knowledge and experience on various aspects of OL and related diseases is necessary. During follow-up, changes in an OL-lesion can be easily detected by the dentist, allowing for timely referral to an OMFS for assessment and, if necessary, further investigation. Several studies confirm the findings in this study on dental students’ and dentists’ knowledge of oral pathology and their practices towards OSCC. A review, including worldwide studies, on the knowledge of dental surgeons on oral and oropharyngeal cancer showed that a good number of dentists had limited knowledge about oral/oropharyngeal cancer, especially in the technical capacity for early detection and that there is a need for constant ongoing education of dentists on the subject.^20^ Canadian dentists reported that 92% of them were sufficiently trained in recognising early signs and symptoms of OSCC, and in Maryland 94% of the surveyed dentists indicated that they were adequately trained to detect oral cancer.^21^^,^^22^ However, a study performed in the UK found that only 37% of dentists felt confident enough to detect oral cancer.^23^ Still, it is possible that one underestimates one's own latent knowledge and that various signs and symptoms, clinical aspects and features of the lesion, and patient characteristics also influence the likelihood of suspecting an OSCC diagnosis. It has been found that head and neck cancer teaching programs showed a considerable variation across European dental schools and development of a unified teaching program suitable for all European dental schools was advised.^24^ Integrating more modules on oral pathology into the dental curriculum could enhance dentists’ knowledge and confidence regarding recognising and possibly diagnosing OL and OSCC.^25^ To carry out regular follow-up of patients with OL, dentists must be willing, prepared, and motivated to perform follow-up procedures; possess sufficient knowledge of this subject; and have sufficient self-confidence in their competence and skills. The question arises as to whether all OLs could be controlled by dentists. Since the risk of MT is high in OLs with moderate or severe epithelial dysplasia, it seems obvious to exclude OL with these histopathological diagnoses, also because in certain cases shortened follow-up intervals are necessary. In contrast, the chance of MT of OLs without dysplasia is low. In the Western world, hyperkeratosis or no epithelial dysplasia in OL ranges between 63% and 87%.^19^^,^^26^, ^27^, ^28^ So at least half of the patients with no dysplasia of OL who undergo periodic dental check-ups might be suitable for regular follow-up of OL by their dentist. Given the incidence of OL, it is not realistic that every dentist should be able to perform regular follow-up of OL. Adequate follow-up should be performed by dentists who have had additional training and as a result have enhanced attention and awareness of oral mucosal diseases. In recent years, there has been an increasing trend towards dental group practices, and within these settings, it is more likely that there are dentists who have differentiated or developed a keen interest in certain areas within dentistry such as oral medicine.^29^^,^^30^ It makes sense to ask those dentists to take care of the follow-up so that they see more patients with OL and therefore gain more experience, and will also have more accessible contact with the OMFS. Patients can easily be referred back or photos may be sent to the OMFS when the clinical aspect of the OL has changed. Although this study focuses on Dutch dentists, the findings could potentially be applied to dentists in those countries where dentistry is regulated.

The present study does have some limitations. Although the number of participants of the present survey was sufficient, the response was not optimal. But with a response rate of 26.9%, it was in line with online surveys in patient and health care professional surveys, and according to Rowley, a response rate of 20% is generally considered acceptable.^31^ As in many surveys, this study probably also involved socially desirable answers. The questions touched on professional knowledge and skills and it can be expected that dentists show their positive side in their answers or have looked up answers.^32^ Furthermore, the survey was sent to a group of Dutch dentists who take part in an intervision program, who may be expected to be more motivated and interested in profession-related topics than the average dentist. Therefore the results of this study may paint a somewhat overly positive picture of reality. On the other hand, with regard to general personal and professional characteristics, the group of dentists in this study formed a reasonably representative reflection of the population of dentists in the Netherlands^33^ However, it was an omission that it was not asked how many patients with OL were followed up by each dentist, but given the prevalence of OL this number would have been very small anyway.

## Conclusion

The lifelong follow-up of OL in hospitals leads to an increasing case load and higher costs of medical care. Referring OL patients with a low risk of MT back to the dentist, after definitive clinical and histopathological diagnosis by an OMFS, will lead to a decreased patient burden and lower healthcare costs. The results of this study show that dentists are willing to perform the follow-up of OL patients, but that a significant proportion of them do not perform optimal clinical practice or that there are gaps in their knowledge in the field of oral mucosal diseases. Given the incidence of OL, it is not necessary that every dentist should be able to perform regular follow-up of OL patients. In large group practices, the follow-up could be carried out by a dentist who has an affinity for oral mucosal diseases. Supported by additional education, it seems that these interested and dedicated dentists can properly fulfil this controlling and detecting role on selected patients with OL.

## Author contributions

A.M. Najim: Conceptualisation; data curation; investigation; methodology; project administration; writing—original draft; writing—review and editing.

J.J.M. Bruers: Data curation; formal analysis; investigation; methodology; visualisation; writing—review and editing.

J.G.A.M. de Visscher: conceptualisation; investigation; methodology; supervision; project administration; writing—original draft; writing—review and editing.

## Conflict of interest

None disclosed.

## Appendix Supplementary materials

Image, application 1

## Footnotes

Supplementary material associated with this article can be found in the online version at doi:10.1016/j.identj.2024.10.021.
