# Supplementary material for: Knowledge, Attitude, and Practice of Dutch Dentists on Oral Leukoplakia and Their Possible Role in Its Follow-Up
Source: Int Dent J. 2025 Jan 3;75(2):1029–35. doi: 10.1016/j.identj.2024.10.021 (PMC11976555; doi:10.1016/j.identj.2024.10.021)
Supplement: Supplementary file 1 [file mmc1.docx]

**Appendix: Supplementary tables**

| Table A Frequency of oral subsites that are checked by dentists during regular dental check-up | | |
| --- | --- | --- |
|  | *never / sometimes* | *usually / always* |
| gingiva | 1.6% | 98.4% |
| cheek | 6.2% | 93.8% |
| vestibule | 6.6% | 93.4% |
| palate | 7.1% | 92.9% |
| tongue | 8.7% | 91.3% |
| lip | 10.5% | 89.5% |
| floor of mouth | 20.6% | 79.4% |
| tonsillar pillar | 65.4% | 34.6% |
| n = 437 | | |

| Tabel B Number of oral subsites that are checked by dentists during regular dental check-up | |
| --- | --- |
| 0 | 0.9% |
| 1 - 2 | 2.3% |
| 3 - 4 | 4.8% |
| 5 - 6 | 18.3% |
| 7 - 8 | 73.7% |
| *mean (sd)* | *6.7 ( 1.5)* |
| n = 437 | |

| Table C Whether or not perform follow-up of patients with OL at request of OMFS by dentists | |
| --- | --- |
| yes | 57.0% |
| no | 43.0% |
| n = 437 | |

| Table D Policy of dentists in case of an oral mucosal lesion without a clear clinical diagnosis | | |
| --- | --- | --- |
| *Right* |  | 32.7% |
| - refer patient in case of an unknown diagnosis | 19.1% |  |
| - refer patient for conformation of suspected diagnosis | 10.8% |  |
| - make a picture and send it for consultation to a specialist | 2.8% |  |
| *Wrong* |  | 67.3% |
| - wait and see a few weeks; if unchanged than refer | 57.9% |  |
| - wait and see a few weeks; if unchanged wait a few weeks again | 7.6% |  |
| - depending on the clinical aspect, direct referral or wait and see | 1.8% |  |
| n = 437 | | |

| Table E Seen own patient with suspicion of oral cancer, recently referred a case to OMFS and whether or not it turned out to be oral cancer according to dentists | | | |
| --- | --- | --- | --- |
| *have seen own patient with suspicion of oral cancer* | *recently case referred with suspicion of oral cancer* | *turned out to be oral cancer* |  |
| no |  |  | 13.3% |
| yes | yes | yes | 62.7% |
| yes | yes | no | 21.3% |
| yes | no | unknown ^#1^ | 2.7% |
| n = 437 | | | |
| *#1 Those who did not immediately refer to OMFS gave following reasons:*  *- I consulted the OMFS and also the treating specialist in geriatric medicine, Mrs. was demented and in her last phase of life, family has chosen not to refer.*  *- To rule out if it was a bite trauma. Patient was not a smoker and did not consume much alcohol.*  *- Patient indicated that he would visit his GP for a second opinion. Despite insistence, the patient did not want to be referred yet.*  *- Wait and see for two weeks; since the mucosal lesion was unchanged the patient was referred to OMFS.*  *- Referral to specialist was made by the periodontist where he was treated.*  *- Clinical diagnosis was not clear so wait and see. After 2 weeks, the lesion was not changed and patient was referred to OMFS.*  *- Wait and see since the patient was very old and sick. Died shortly afterwards.*  *- t's been years ago, but in my memory I saw the patients after 2 weeks and was then referred.*  *- The patient did not want to be referred.*  *- Could also be a bite trauma; patient couldn't remember a trauma. Had a consultation with OMFS. Wait and see; a few days later, the lesion was already reduced in size and disappeared after 2 weeks.*  *- It did not seem to be a lesion that was not suspicious for cancer. The patient visited another dentists who referred the patient immediately.*  *- Wait and see for two weeks.*  *- Wait and see for three weeks.* | | | |

| Table F Degree of certainty of dentists of the diagnosis oral cancer in most recent case who was referred to OMFS | | |
| --- | --- | --- |
|  | *turned out to be oral cancer* | |
|  | *yes* | *no* |
| (very) uncertain | 4.4% | 35.5% |
| not uncertain, not certain | 15.3% | 48.4% |
| (very) certain | 80.3% | 16.1% |
| n | 274 | 93 |
| *Chi-Square = 131.436 / df = 2 / p < 0.001* | | |

| Table G Description that best fits the term oral epithelial dysplasia according to dentists | |
| --- | --- |
| reversible change from one cell type to another cell type | 13.6% |
| presence of abnormal cells* | 84.3% |
| increased cell volume | 2.1% |
| decreased cell volume | 0.0% |
| n = 381  **Deemed the correct answer to the question* | |

| Table H Correct answer of dentists to the statements about OL | |
| --- | --- |
| leukoplakia of the floor of the mouth and tongue show a higher risk of malignant transformation than leukoplakia of the palate and gingiva.* | 73.9% |
| there is no difference in the risk of malignant transformation of leukoplakia of the floor of the mouth, tongue, palate and gingiva. | 26.1% |
| n = 380  **Deemed the correct answer to the question* | |

| Table I Degree of certainty of dentists about various statements | | | |
| --- | --- | --- | --- |
|  | *(very) uncertain* | *not uncertain / certain* | *(very) certain* |
| a | The quality of education on oral mucosa diseases was good during my dental study | | |
|  | 16.7% | 33.7% | 49.6% |
| b | Received sufficient education about premalignant oral lesions during my dental study | | |
|  | 21.9% | 32.4% | 45.7% |
| c | Know what oral leukoplakia is | | |
|  | 5.0% | 24.5% | 70.5% |
| d | Know the importance of the histopathological diagnosis of leukoplakia (hyperkeratosis/dysplasia) for the follow-up policy | | |
|  | 15.9% | 31.9% | 52.2% |
| e | Confident to take over follow-up of all patients with leukoplakia irrespective of the histopathological diagnosis | | |
|  | 50.1% | 30.8% | 19.1% |
| f | Confident to take over follow-up of patients with leukoplakia with the histopathological diagnosis hyperkeratosis or mild dysplasia of the tongue and floor of the mouth | | |
|  | 47.8% | 35.2% | 17.0% |
| g | Confident to take over follow-up of patients with leukoplakia of the gingiva with the histopathological diagnosis hyperkeratosis | | |
|  | 34.5% | 30.0% | 35.5% |
| h | If I take over the follow-up of patients with leukoplakia, I will also show the patient to a fellow dentist in the practice | | |
|  | 21.7% | 22.5% | 55.9% |
| i | If I take over the follow-up of patients with leukoplakia, I do need further education about oral mucosal diseases | | |
|  | 4.7% | 15.4% | 79.9% |
| j | Leave the follow-up of patients with leukoplakia to a colleague in the practice who has experience and affinity with the disease | | |
|  | 55.4% | 34.2% | 10.4% |
| k | All patients with leukoplakia should be checked by a OMFS | | |
|  | 32.6% | 30.3% | 37.1% |
| l | Received sufficient education about various aspects of oral cancer during my dental study | | |
|  | 21.9% | 33.9% | 44.1% |
| m | Can recognize oral cancer | | |
|  | 16.2% | 42.3% | 41.5% |
| n = 383 | | | |

| Table J Confidence of dentists on gathered knowledge and received education about oral mucosal diseases, leukoplakia and oral cancer: sum score of statement a, b, c, d, l and m from table I | |
| --- | --- |
| very uncertain (score 1 - 9) | 0.8% |
| uncertain (score 10 - 15) | 11.0% |
| not uncertain, not certain (score 16 - 21) | 45.4% |
| certain (score 22 - 27) | 39.9% |
| very certain (score 28 - 30) | 2.9% |
| *mean (sd)* | *20.5 (3.9)* |
| *median* | *21.0* |
| *mode* | *23.0* |
| *minimum* | *8.0* |
| *maximum* | *30.0* |
| n = 383 | |
| *Crohnbach’s Alpha = 0.798* | |

| Table K Confidence of dentists to take over the follow-up of patients with OL at different subsites and various histopathological diagnosis: sum score of statement e, f, and g from table I | |
| --- | --- |
| very uncertain (score 1 - 4) | 8.6% |
| uncertain (score 5 - 7) | 32.6% |
| not uncertain, not certain (score 8 – 10) | 38.6% |
| certain (score 11 - 13) | 18.3% |
| very certain (score 14 – 15) | 1.8% |
| *mean (sd)* | *8.2 (2.7)* |
| *median* | *9.0* |
| *mode* | *6.0* |
| *minimum* | *3.0* |
| *maximum* | *15.0* |
| n = 383 | |
| *Crohnbach’s Alpha = 0.889* | |
